# Construction of pH-responsive hydrogel coatings on titanium surfaces for antibacterial and osteogenic properties

**DOI:** 10.3389/fchem.2025.1546637

**Published:** 2025-02-19

**Authors:** Shan Peng, Yueru Liu, Wei Zhao, Xinpeng Liu, Ronghua Yu, Yonglin Yu

**Affiliations:** ^1^ Department of Pathology, Affiliated Hospital of Zunyi Medical University, Zunyi, Guizhou, China; ^2^ Department of Pathology, Zunyi Medical University, Zunyi, Guizhou, China

**Keywords:** titanium, chitosan, polydopamine, antimicrobial peptides, biocompatibility, antibacterial activity, osteogenic ability

## Abstract

Infection is one of the leading causes of failure in titanium-based implant materials during clinical surgeries, often resulting in delayed or non-union of bone healing. Furthermore, the overuse of antibiotics can lead to bacterial resistance. Therefore, developing a novel titanium-based implant material with both antimicrobial and osteogenic properties is of great significance. In this study, chitosan (CS), polydopamine (PDA), and antimicrobial peptides (AMPs) HHC36 were applied to modify the surface of titanium, resulting in the successful preparation of the composite material Ti-PDA-CS/PDA@HHC36 (abbreviated as T-P-C/P@H). CS promotes osteogenesis and cell adhesion, providing an ideal microenvironment for bone repair. PDA enhances the material’s biocompatibility and corrosion resistance, offering cell adhesion sites, while both components exhibit pH-responsive characteristics. The HHC36 effectively prevents infection, protecting the bone repair material from bacterial damage. Overall, the synergistic effects of these components in T-P-C/P@H not only confer excellent antimicrobial and osteogenic properties but also improve biocompatibility, offering a new strategy for applying titanium-based implants in clinical settings.

## 1 Introduction

Bone injuries caused by trauma, bone tumors, or inflammation are common clinical conditions (Wei et al., 2022). Clinical treatment often involves artificial bone grafts, bioactive substances, and stem cells to accelerate healing. Titanium-based implant materials, known for their excellent corrosion resistance, mechanical properties, and biocompatibility, are widely used in fields such as orthopedics, dentistry, and plastic surgery (Jiang et al., 2023; Villegas et al., 2024). However, titanium-based implants still face challenges in clinical applications, such as poor bone integration and bacterial infections, often leading to implant failure (Wang Z. et al., 2021; Tang et al., 2024). In response to bacterial infections, commonly used antibiotic treatments can exacerbate bacterial resistance, and secondary procedures such as implant revision or infection site removal can impose significant burdens on both physicians and patients (Chen et al., 2021). Therefore, surface modification of titanium-based implant materials to develop implants with both antimicrobial and osteogenic properties is of profound significance in improving patient outcomes and reducing the economic burden (Yang Z. et al., 2021; Zhang et al., 2022; Wang S. et al., 2023).

Recent studies have shown that both “defensive” antimicrobial strategies to inhibit bacterial adhesion and “offensive” antimicrobial strategies to kill bacteria require the construction of specific interfaces on the surface of titanium-based implants. These interfaces must have properties such as anti-adhesion, antimicrobial agent release, and osteogenesis promotion (Wu et al., 2021; Nikoomanzari et al., 2022; Han et al., 2023). Hydrogels, with their tunable properties, are capable of meeting specific requirements under varying conditions. In aqueous environments, hydrogels swell and provide a cell-extracellular matrix-like microenvironment. They are also easily loaded with drugs and bioactive factors, promoting cell migration, adhesion, proliferation, and differentiation. As a result, hydrogels are widely used as scaffold materials in tissue engineering and are ideal for surface modification of titanium-based implants (Tang et al., 2023; Zhang et al., 2023; Zhang L. et al., 2024). Hydrogels are three-dimensional crosslinked networks made from hydrophilic homopolymers, copolymers, or macromolecules and have found broad applications in biomedical fields, including physiological and pathological mechanism studies, tissue regeneration, and disease treatment (Correa et al., 2021; Dong and Guo, 2021; Liu J. et al., 2021). Hydrogels are typically synthesized from natural polymers (such as gelatin, hyaluronic acid, chitosan, collagen, and alginate) and synthetic polymers (such as polyethylene oxide and polyacrylamide) (Zhang X. et al., 2024). Among these, CS, derived from the deacetylation of chitin, has excellent biocompatibility, biodegradability, antimicrobial properties, and moisture retention, making it widely applied in biomedical fields (Lin et al., 2021; Mottaghitalab et al., 2024). CS’s molecular structure resembles glycosaminoglycans (GAGs), which are key components of the extracellular matrix that connect with collagen fibers, thereby offering a conducive microenvironment for extracellular matrix formation and cell proliferation, with potential osteogenic effects (Wang et al., 2018). Moreover, CS’s amino groups can be protonated in mildly acidic conditions, enabling pH responsiveness (Zhang et al., 2016). Chemical modifications such as carboxymethylation, acylation, and alkylation can enhance CS’s stability, water solubility, membrane permeability, and targeted drug delivery capabilities (An et al., 2024). Despite its significant biomedical potential, CS’s low mechanical properties limit its application in bone tissue engineering, which is why it is often used in combination with other bioactive materials (Xu et al., 2021; Liu et al., 2023). Recently, PDA, due to its functional groups such as amines and catechols in its structure, has demonstrated excellent properties, including wet adhesion, antimicrobial activity, free radical scavenging, UV shielding, photothermal conversion, and biocompatibility. As a result, PDA has found widespread application in chemistry, biology, and medicine, particularly in the biomedical field (Fu et al., 2021; Yang P. et al., 2021). Studies have shown that PDA’s adhesion mechanism depends on interactions with various substrates through metal coordination, Michael addition, Schiff base reactions, and hydrogen bonding, allowing surface functionalization and modulation of biological effects (Hemmatpour et al., 2023; Battaglini et al., 2024). The amino and hydroxyl groups in CS can interact with the phenolic hydroxyl and amine groups in PDA through hydrogen bonding or electrostatic interactions, promoting PDA-CS interaction and further enhancing bioactivity (Jin et al., 2020). PDA combined with osteogenic factors can be used to improve implant osseointegration and promote bone formation (Wang L. et al., 2021; Dimassi et al., 2022; Wan et al., 2022; Wu et al., 2023). Although CS and PDA have some antimicrobial effects on titanium-based implants, they cannot achieve complete bacterial eradication. Antibiotics, as traditional antimicrobial agents, have greatly reduced bacterial infections since the discovery of penicillin in 1928, saving countless lives (Davies and Davies, 2010). While antibiotic development has made significant progress, its widespread use and misuse have accelerated the development of bacterial resistance (de la Fuente-Nunez et al., 2023). Compared to traditional antibiotics, AMPs offer distinct advantages, such as slower development of resistance, broad-spectrum activity against biofilms, and the ability to modulate host immune responses. AMPs are an integral part of innate immunity in humans and other higher organisms (Magana et al., 2020; Deo et al., 2022). AMPs typically consist of 10–100 amino acid residues and can be classified into natural and synthetic types. They exhibit broad-spectrum antibacterial activity and low toxicity to eukaryotic cells, making them a popular research direction for antimicrobial agents (Zhang et al., 2021; Xuan et al., 2023). However, the clinical application of AMPs faces challenges, as infected tissues often possess strong proteolytic activity, making it difficult for AMPs to survive in such environments. Furthermore, systemic administration of AMPs presents poor stability and a short plasma half-life. Therefore, improving the bioavailability or stability of AMPs through delivery systems or formulations is key to their clinical application (Wang C. et al., 2021). HHC36 (KRWWKWWRR), a highly efficient AMP designed through artificial neural network prediction, contains nine amino acids and demonstrates potent antibacterial activity against multiple antibiotic-resistant “superbugs”, such as methicillin-resistant *Staphylococcus aureus* (MRSA). It exhibits better antibacterial performance than traditional antibiotics (e.g., ciprofloxacin, cefotaxime) and clinical candidate AMPs (e.g., MX226 and hLF1-11) (Gao et al., 2023; Wang B. et al., 2023; Dong et al., 2024). Additionally, studies have shown that biomaterials loaded with HHC36 display enhanced antibacterial properties (Chen et al., 2020; Sandhu et al., 2022).

**FIGURE 1:**
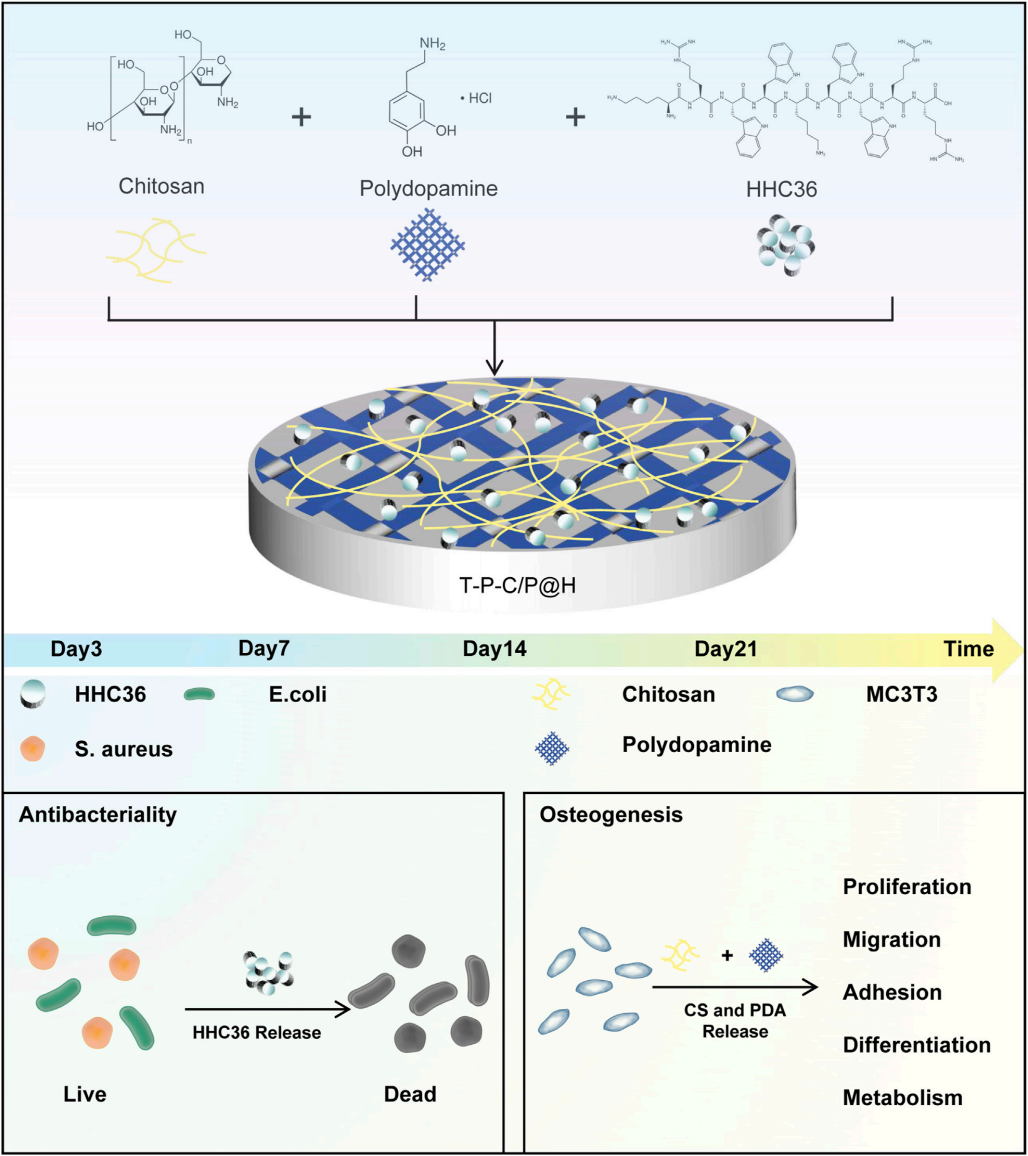
Schematic illustration of the composite coating preparation process and its antibacterial and osteogenic properties.

This study aims to design a titanium-based implant material with antimicrobial and osteogenic properties. By modifying the titanium surface with CS, PDA, and the HHC36, a pH-responsive composite material, T-P-C/P@H, was successfully developed (Figure 1). Under normal physiological conditions, T-P-C/P@H effectively shields the low toxicity of AMPs, providing excellent biocompatibility and anti-adhesion properties, while demonstrating osteogenic potential. In the event of bacterial infection, T-P-C/P@H responds sensitively to changes in the local pH environment, rapidly releasing HHC36 to eradicate bacteria. This responsive antimicrobial and osteogenic composite system offers an innovative approach to the development of new, efficient implantable medical devices.

## 2 Materials and methods

### 2.1 Materials

The Northwest Institute for Nonferrous Metal Research (Xi’an, China) purchased the titanium sheets and spikes. Chitosan and dopamine hydrochloride were purchased from Sigma-Aldrich (United States). Tris (tris (hydroxymethyl) aminomethane) was purchased from Coolaber (Beijing, China). The SuperKine™ Ultra-sensitive Cell Proliferation Detection Kit (CCK-8) (catalog number: BMU106) was purchased from Abbkine (Wuhan, China). The Calcein/PI Cell Viability and Cytotoxicity Assay Kit (catalog number: C2015S), Alkaline Phosphatase Assay Kit (catalog number: P0321S), BCIP/NBT Alkaline Phosphatase Color Development Kit (catalog number: C3206), Osteoblast Mineralization Staining Kit (Alizarin Red S method) (catalog number: C0148S), and Bacterial Live/Dead Staining Kit (DMAO/PI) (catalog number: C2030S) were purchased from Beyotime (Shanghai, China). The BCA Protein Assay Kit (catalog number: PC0020) and the Alizarin Red S Staining Quantification Kit (CPC method) (catalog number: G3283) were purchased from Solarbio (Beijing, China). The MC3T3-E1 Subclone 14 cells (mouse calvarial osteoblasts, STR-verified) were purchased from Pricella (catalog number: CL-0378) (Wuhan, China). The MicroRNA Dual Column Kit was purchased from Magen (catalog number: R4114) (Guangzhou, China). The PrimeScript™ RT Reagent Kit (Perfect Real Time) (catalog number: RR037A) and the TB Green^®^ Premix Ex Taq™ II (Tli RNaseH Plus) high-specificity qPCR reagent (catalog number: RR820A) were sourced from Takara (Beijing, China). The antimicrobial peptide HHC36 was purchased from Hubei Qiangyao Biotechnology Co., Ltd. (Hubei, China).

### 2.2 Material preparation and grouping

The titanium sheets were polished using sandpaper with grits ranging from 600 to 2000 and then subjected to ultrasonic cleaning in ethanol and distilled water to remove surface contaminants. After cleaning, the samples were sterilized using high-pressure steam and allowed to dry, resulting in the control group Ti. The Ti sheets were then immersed in a 2 mg/mL dopamine solution prepared in a 10 mM Tris buffer (pH 8.5) and shaken at 37°C and 150 rpm for 24 h to obtain Ti-PDA (abbreviated as T-P). Next, CS/PDA hydrogel was uniformly coated onto the T-P surface using a spin coater, with 0.002 g of PDA dissolved in 1 mL of Tris solution (10 mM, pH 8.5) and 0.02 g of CS dissolved in 1 mL of acetic acid solution (0.1 M), ensuring thorough mixing at room temperature to obtain Ti-PDA-CS/PDA (abbreviated as T-P-C/P). Subsequently, CS/PDA@HHC36 hydrogel was similarly applied to the T-P surface, with 0.002 g of PDA dissolved in 1 mL of Tris solution (10 mM, pH 8.5) and 0.02 g of CS dissolved in 1 mL of HHC36 (5 mg/mL) acetic acid solution (0.1 M), also mixed at room temperature thoroughly to yield T-P-C/P@H. The spin coater was operated at 6,500 rpm for 30 s to apply 40 μL of both CS/PDA hydrogel and CS/PDA@HHC36 hydrogel onto the titanium sheets, repeating this process five times to ensure a uniform coating on the Ti surface. All samples intended for biological experiments were sterilized by ultraviolet irradiation for 1 hour.

### 2.3 Preparation of extract

Based on the surface area of the titanium sheets, the sheets and the calculated amount of culture medium were added to a 6-well plate and incubated in a 37°C, 5% CO₂ incubator for 72 h. The extract was then filtered through a 0.22 μm pore size filter to sterilize and stored for later use.

### 2.4 Characterization

First, scanning electron microscopy (SEM, TESCAN MIRA4 LMH) and energy-dispersive X-ray spectroscopy (EDS, Oxford Instruments Ultim Max 40) were employed to capture surface morphology images and elemental distribution spectra. Next, the wettability of the material surfaces was assessed using a contact angle goniometer (Biolin Theta Flex), with contact angles recorded via digital photography. The elemental composition of the materials was analyzed using X-ray photoelectron spectroscopy (XPS, Shimadzu AXIS SUPRA+). Lastly, atomic force microscopy (AFM, Bruker Edge) was used to evaluate surface roughness, providing further insight into surface characteristics.

### 2.5 *In vitro* release of HHC36

The T-P-C/P@H materials were immersed in 2 mL of phosphate-buffered saline (PBS) at pH 5, 5.5, 6, and 7.4. The samples were incubated at 37°C with constant shaking at 30 rpm/min for different time intervals (12, 24, 48, 72, 96, 120, 144, 168, 192, and 216 h). At each time point, 0.2 mL of the solution was collected, and fresh PBS was added to maintain a constant total volume. After the final collection, the HHC36 content in the supernatant was measured using the BCA protein assay kit. Absorbance was recorded at 562 nm using a full-wavelength microplate reader (Thermo Fisher, Multiskan Sky). HHC36 concentration was calculated using a standard curve. The cumulative release amount of HHC36 was determined using the following formula: Cumulative Release (%) = m (V0 × Ct + V × ∑Ci) × 100%/m. Where: V0 is the total volume of the release medium; Ct is the drug concentration at the final sampling time point; V is the volume sampled at each time point; ∑Ci is the sum of drug concentrations at all sampling time points; m is the total amount of drug in the formulation (in mg).

### 2.6 CCK-8 assay

Cell proliferation activity of MC3T3-E1 Subclone 14 (MC3T3-E1) in response to Ti, T-P, T-P-C/P, and T-P-C/P@H materials was assessed using the SuperKine™ Cell Counting Kit 8 (CCK-8) assay. MC3T3-E1 cells were seeded in a 96-well plate at a density of 2 × 10³ cells per well and incubated at 37°C with 5% CO₂ for 24 h. After incubation, extract solutions from the various materials were added to the wells. At 24, 48, and 72 h post-incubation with the extract solutions, CCK-8 reagent was added to each well to assess cell viability. The optical density (OD) at 450 nm was measured using a full-wavelength microplate reader. The OD values from the experimental or control groups were normalized by subtracting the blank group values, and the resulting data were plotted against time to analyze the effect of the materials on cell viability.

### 2.7 Live/dead cell staining assay

The effects of Ti, T-P, T-P-C/P, and T-P-C/P@H materials on MC3T3-E1 cells were assessed using live/dead cell staining. MC3T3-E1 cells were seeded in a 96-well plate at a density of 2 × 10³ cells per well and incubated at 37°C with 5% CO_2_ for 24 h. After incubation, extract solutions from the various materials were added to the wells. Staining was performed at 24, 48, and 72 h using the Calcein/PI Cell Viability and Cytotoxicity Assay Kit. Images were captured using an inverted fluorescence microscopy system (Leica, DMi8 + cooled + DFC7000). This allowed for the observation and recording of cell viability and death at different time points, providing insights into the biocompatibility of the materials with MC3T3-E1 cells.

### 2.8 Cell migration ability

The scratch assay was used to assess the effects of Ti, T-P, T-P-C/P, and T-P-C/P@H materials on the migration ability of MC3T3-E1 cells. MC3T3-E1 cells were seeded in a 6-well plate at a density of 1.2 × 10⁵ cells per well and incubated at 37°C with 5% CO₂ for 24 h until the cells reached 90% confluence. A 10 μL pipette was then used to create a uniform scratch in the center of each well, followed by washing with PBS to remove any floating cells. Serum-free extract solutions of each material were subsequently added to the wells. The migration of cells was photographed using an inverted microscope imaging system (Leica, DMi1 + C1) at 0, 12, and 24 h, to assess the influence of different materials on cell migration ability.

### 2.9 Plate counting assay

The antibacterial properties of Ti, T-P, T-P-C/P, and T-P-C/P@H materials were assessed using a plate counting method, with *Staphylococcus aureus* (SA) and *Escherichia coli* (*Escherichia coli*) as representative bacterial strains. Titanium sheets of each material were placed in 24-well plates and incubated with bacterial suspensions for 24 h. After incubation, the suspension was discarded, and the wells were washed with 1 mL of PBS to remove any non-adherent bacteria. Following this, 1 mL of PBS was added again, and the wells were subjected to ultrasonic agitation for 5 min to detach the bacteria adhered to the material surfaces. The resulting bacterial suspensions were then diluted to appropriate concentrations and spread evenly on agar plates. These plates were incubated at 37°C for 24 h. Finally, images of the agar plates were captured, and colony counts were performed to evaluate the antibacterial efficacy of the materials.

### 2.10 Inhibition zone assay

The antibacterial properties of the Ti, T-P, T-P-C/P, and T-P-C/P@H materials were assessed through an inhibition zone assay, using SA and *E. coli* as representative bacterial strains. Titanium sheets from each group were placed on agar plates uniformly inoculated with the bacterial suspensions. The plates were then incubated at 37°C for 24 h. After incubation, by observing the size of the inhibition zones to assess the antibacterial efficacy, the results were documented with photographs.

### 2.11 Live/dead bacterial staining

The antibacterial properties of Ti, T-P, T-P-C/P, and T-P-C/P@H materials were assessed through live/dead bacterial staining using SA and *E. coli* as representative strains. First, material sheets were incubated with bacterial suspensions in 24-well plates at 37°C for 24 h. After incubation, the bacterial suspensions were stained according to the protocol of a live/dead bacterial staining kit (DMAO/PI). Finally, the stained samples were observed and photographed using an upright fluorescence imaging system (Leica DM3000) to visually assess the antibacterial effects of the materials by distinguishing between live and dead bacteria.

### 2.12 Alkaline phosphatase (ALP) staining and quantitative assay

The effect of Ti, T-P, T-P-C/P, and T-P-C/P@H materials on the osteogenic potential of MC3T3-E1 cells was assessed using both ALP staining and quantitative assays. MC3T3-E1 cells were seeded at a density of 3 × 10⁴ cells per well in 24-well plates and incubated under 37°C with 5% CO₂ for 24 h. After incubation, the extract solutions of each material were added, and the culture medium was replaced every other day. On days 4 and 7, ALP activity was assessed using the BCIP/NBT ALP staining kit and images were captured using an inverted microscope. In parallel, ALP activity was quantitatively measured using an ALP assay kit, with absorbance recorded at 405 nm using a full-wavelength microplate reader. ALP activity was calculated by comparing the absorbance between the experimental and control groups, following the enzyme activity definition.

### 2.13 Mineralization staining and quantitative analysis

The effect of Ti, T-P, T-P-C/P, and T-P-C/P@H materials on the osteogenic potential of MC3T3-E1 cells was assessed through Alizarin Red S staining and quantitative analysis of mineralized nodules. MC3T3-E1 cells were seeded at a density of 3 × 10^4^ cells per well in a 24-well plate and incubated at 37°C with 5% CO_2_ for 24 h. After incubation, the respective extract solutions of each material were added, and the culture medium was replaced every other day. On days 14 and 21, mineralized nodules were stained using the Alizarin Red S staining kit and images were captured with an inverted microscope. In parallel, quantitative analysis of the mineralized nodules was performed using the Alizarin Red S staining kit (CPC method). Absorbance was measured at 560 nm using a full-wavelength microplate reader, and the mineralized calcium content was calculated based on a standard curve.

### 2.14 Assessment of osteogenic-related genes by using quantitative real-time polymerase chain reaction (qRT-PCR)

The effects of Ti, T-P, T-P-C/P, and T-P-C/P@H materials on osteogenic-related genes (ALP, Runx2, OPN, OCN) in MC3T3-E1 cells were assessed using qRT-PCR. MC3T3-E1 cells were seeded at a density of 1.2 × 10⁵ cells per well in a 6-well plate and incubated at 37°C with 5% CO₂ for 24 h. Following this, the extracts of the respective materials were added, and the culture medium was changed every other day. The cell status was monitored daily using an inverted microscope. On days 7 and 14 after the addition of the extracts, RNA was extracted using the microRNA extraction kit, and reverse transcription was performed using PrimeScript™ RT Reagent Kit (Perfect Real Time). qRT-PCR was conducted with TB Green^®^ Premix Ex Taq™ II (Tli RNaseH Plus). Reverse transcription reactions were performed using a gradient PCR machine (Bio-Rad, T100), and real-time PCR was carried out using a fluorescence quantitative PCR machine (Bio-Rad, CFX96 Touch). The results were analyzed using the 2^(-∆∆CT)^ method. The primer sequences for the target genes are listed in Table 1.

**TABLE 1:** Primer Sequences used in qRT-PCR.

Gene	Forward primers (5′to 3′)	Reverse primers (5′to 3′)
β-actin	TAT​GCT​CTC​CCT​CAC​GCC​ATC​C	GTC​ACG​CAC​GAT​TTC​CCT​CTC​AG
ALP	TGA​CTA​CCA​CTC​GGG​TGA​ACC	TGA​TAT​GCG​ATG​TCC​TTG​CAG
OPN	AGA​GCG​GTG​AGT​CTA​AGG​AGT	TGC​CCT​TTC​CGT​TGT​TGT​CC
OCN	GACCGCCTACARACGCATCTA	GCA​GAG​AGA​GAG​GAC​AGG​GAG
Runx2	TTA​GGC​AGG​GCC​AAC​AAG​AG	AGC​CAC​ACT​TAG​GGA​TTG​GC

### 2.15 Statistical analysis

All experiments were performed at least three times. The data were statistically analyzed using one-way analysis of variance (ANOVA) using GraphPad Prism 10 software (GraphPad Software, San Diego, CA, United States). *p* < 0.05 indicates a statistically significant difference.

## 3 Results and discussion

### 3.1 Characterization of T-P-C/P@H

**FIGURE 2:**
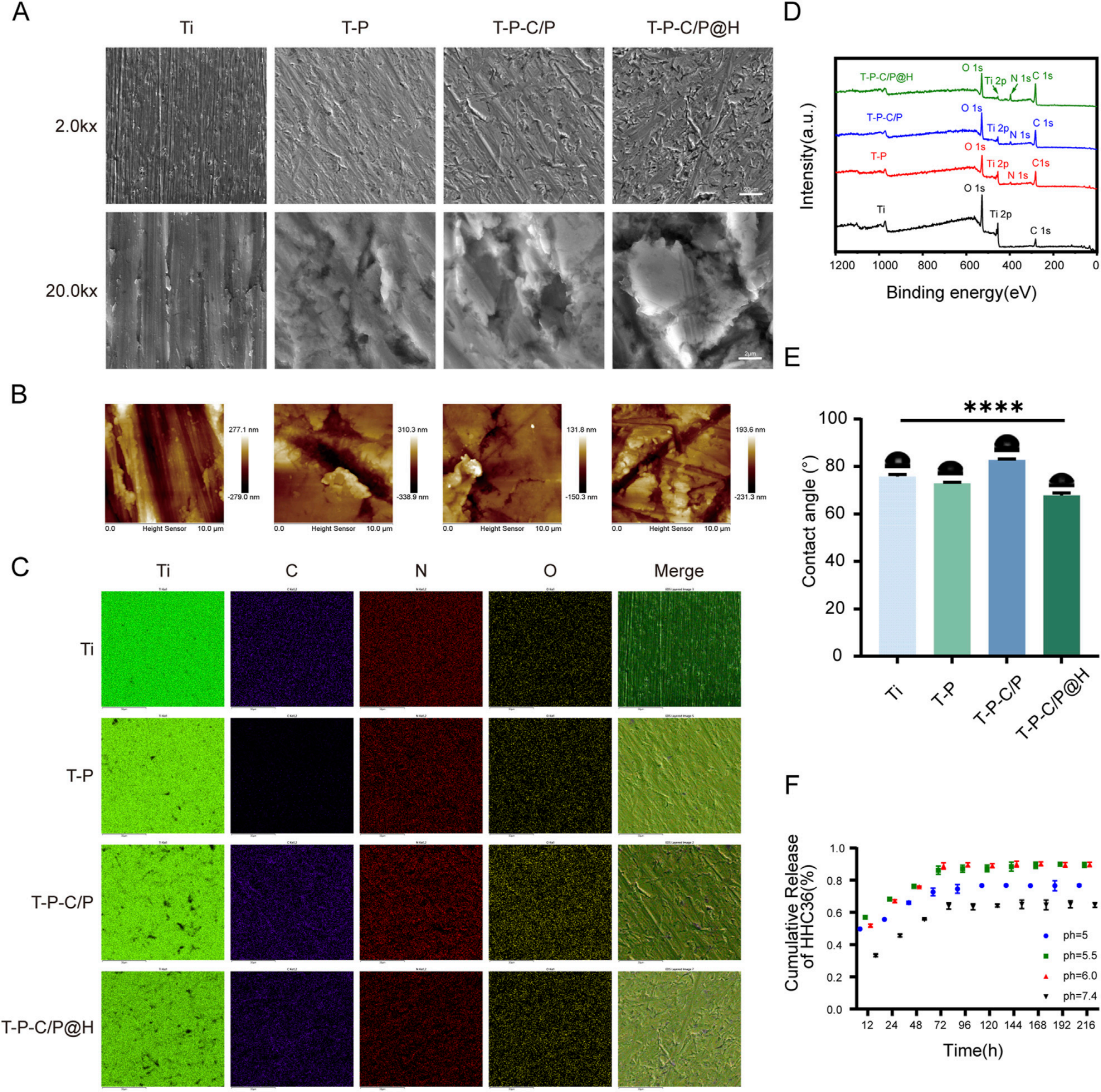
Characterization of T-P-C/P@H. **(A)** SEM images of Ti, T-P, T-P-C/P, and T-P-C/P@H (upper scale bars = 20 μm, lower scale bars = 2 μm). **(B)** AFM 2D-morphology images of Ti, T-P, T-P-C/P, and T-P-C/P@H (Scale bar = 10 μm). **(C)** EDS mapping of Ti, T-P, T-P-C/P, and T-P-C/P@H (Scale bars = 50 μm). **(D)** XPS survey spectra of Ti, T-P, T-P-C/P, and T-P-C/P@H. **(E)** Water contact angles of Ti, T-P, T-P-C/P, and T-P-C/P@H. **(F)** The release profile of HHC36 in PBS at different pH levels up to 9 days. Data are expressed as mean ± SD (n = 3); *p < 0.05, **p < 0.01, ***p < 0.001, ****p < 0.0001, compared with the Ti group.

The surface morphology and roughness of Ti, T-P, T-P-C/P, and T-P-C/P@H materials were characterized using SEM and AFM (Figures 2A, B). The Ti surface exhibited prominent, visible scratches due to the polishing treatment. After alkaline treatment with PDA-Tris solution (pH = 8.5), the roughness of T-P (91.5 nm) increased slightly compared to Ti (87.0 nm). The T-P-C/P material, after the uniform spraying of CS/PDA hydrogel, formed a smooth polymer coating that covered the surface scratches, resulting in a lower roughness (33.1 nm). T-P-C/P@H, which incorporates the antimicrobial peptide HHC36 onto the T-P-C/P surface, showed a slight increase in roughness (62.3 nm). EDS analysis revealed that the elemental composition of T-P-C/P@H included Ti, O, N, and C (Figure 2C). The content of C, N, and O gradually increased as these materials were sequentially loaded onto the titanium surface (Supplementary Figure S1). Furthermore, XPS analysis of different samples confirmed that, after loading CS, PDA, and HHC36, the Ti content on the surface of Ti decreased. In contrast, the C, N, and O contents increased (Figure 2D). The high-resolution XPS spectra of C1s, O1s, and N1s for Ti, T-P, T-P-C/P, and T-P-C/P@H are shown in Supplementary Figure S2–S4. In the O1s spectra, titanium-oxygen bonds (Ti-O) were detected in all samples, indicating the presence of titanium. Oxygen vacancies (Ov) were also observed in all samples, which may influence the materials’ chemical properties and reactivity. The presence of phenolic hydroxyl groups (-OH) in the T-P confirms the successful coating of PDA on the Ti surface (Tan et al., 2021). In T-P-C/P, the increased intensity of the hydroxyl (-OH) related peaks suggests the successful incorporation of CS (Alqarni et al., 2024). In the N1s spectra, the detection of amide groups (-CONH-) and carbon-nitrogen bonds (C-N) in T-P-C/P@H indicates the successful incorporation of HHC36 (Liu K. et al., 2021). After alkaline treatment, the water contact angle of Ti decreased from 76.66° to 72.24°, indicating that the PDA-Tris solution (pH = 8.5) enhanced the hydrophilicity of T-P. However, the modification with CS/PDA hydrogel increased the contact angle of T-P-C/P to 82.99°, creating a hydrophobic surface. The addition of the hydrophilic antimicrobial peptide HHC36 further improved the hydrophilicity of T-P-C/P@H (Figure 2E). In conclusion, surface modification can influence the surface morphology, roughness, chemical composition, and hydrophilicity of titanium-based materials. These changes may positively impact their biocompatibility, antimicrobial properties, and osteogenic potential. By employing different surface modification techniques, the surface characteristics of titanium-based materials can be tailored to optimize their performance in biomedical applications.

### 3.2 *In Vitro* release of HHC36

Figure 2F shows the release profile of HHC36 from T-P-C/P@H at different pH levels, simulating the acidic microenvironment produced by bacterial metabolism during the early stages of infection. At pH 5.5 and 6.0, HHC36 exhibited rapid release within the first 72 h, followed by a slower, sustained release over approximately 1 week. This dual-phase release allows for an initial burst to control infection and a prolonged release to prevent potential reinfection, indicating a pH-responsive characteristic. This behavior can be attributed to structural changes in T-P-C/P@H under acidic conditions, the surface -NH_2_ of T-P-C/P@H was protonated to form -NH_3_⁺ in an acidic environment, triggering structural alterations that facilitated the release of HHC36 (Fu et al., 2021).

### 3.3 *In vitro* biocompatibility assessment

**FIGURE 3:**
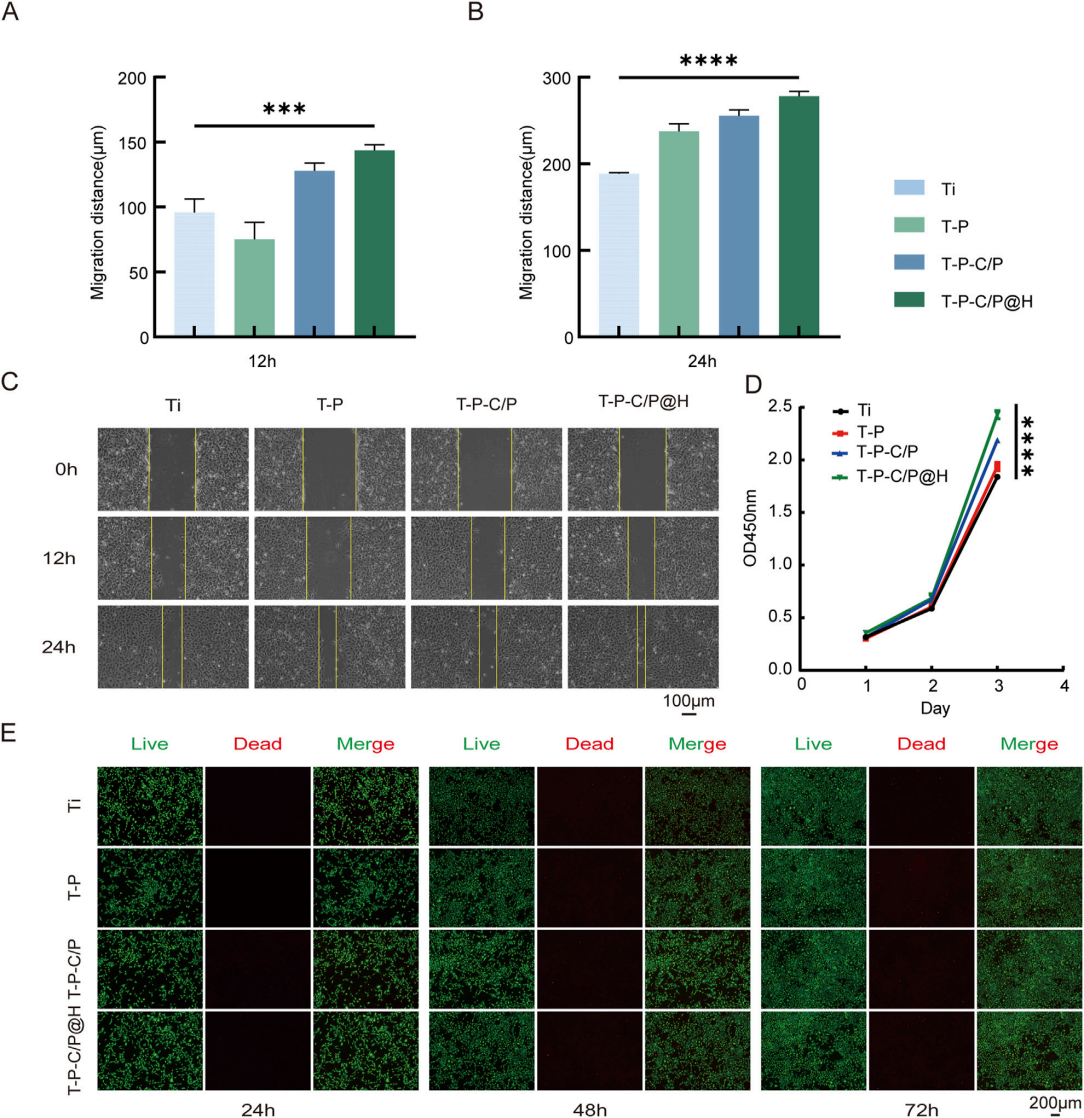
Cell biocompatibility and proliferation of T-P-C/P@H. **(A, B)** Migration distance of MC3T3-E1 cells with different extract treatments for 12 h and 24 h. **(C)** The images of MC3T3-E1 cells at different time points after the scratching with different extract treatments (Scale bar = 100 μm). **(D)** CCK-8 assay for the proliferation of MC3T3-E1 cells after the different extract treatments for 24 h, 48 h, and 72 h. **(E)** Live/Dead assay of MC3T3-E1 cells cultured with different extract treatments for 24 h, 48 h, and 72 h (Green fluorescence: live cells; Red fluorescence: apoptotic or dead cells. Scale bar = 200 μm). Data are expressed as mean ± SD (n = 3); *p < 0.05, **p < 0.01, ***p < 0.001, ****p < 0.0001, compared with the Ti group.

The CCK-8 assay, with the OD value of the experimental or control group minus the OD value of the Ti group, is plotted on the y-axis and time points on the x-axis. As shown in Figure 3D, the OD450 values of T-P, T-P-C/P, and T-P-C/P@H were significantly higher than those of Ti, with statistically significant differences. This indicates that these surface modification strategies not only exhibit no evident cytotoxicity toward Ti but also promote the proliferation of MC3T3-E1 cells to varying degrees, with T-P-C/P@H showing the most pronounced effect, followed by T-P-C/P and T-P. Furthermore, the effect of these modifications on cells was validated using live/dead cell staining. Calcein-AM (green) and PI (red) were used to label live and dead cells, respectively. As illustrated in Figure 3E, Ti, T-P, T-P-C/P, and T-P-C/P@H demonstrated minimal cytotoxicity, with very few dead cells observed. Over 24, 48, and 72 h of culture, the modified materials effectively promoted cell proliferation, consistent with the CCK-8 results. As shown in Figure 3C, the results of the scratch assay indicate that at 12 and 24 h, the number of cells migrating into the scratch area in the Ti, T-P, and T-P-C/P is significantly lower than that in the T-P-C/P@H. Migration distances of the four groups of cells at 0, 12, and 24 h were measured using Adobe Photoshop (Adobe Systems, United States), and statistical analysis was performed using GraphPad Prism (GraphPad Software, United States) (Figures 3A, B). The results demonstrate that cells in the T-P-C/P@H exhibit the highest migration ability, followed by the T-P-C/P, while the migration ability of the T-P is relatively moderate. These differences are statistically significant.

### 3.4 *In vitro* antibacterial ability assessment

**FIGURE 4:**
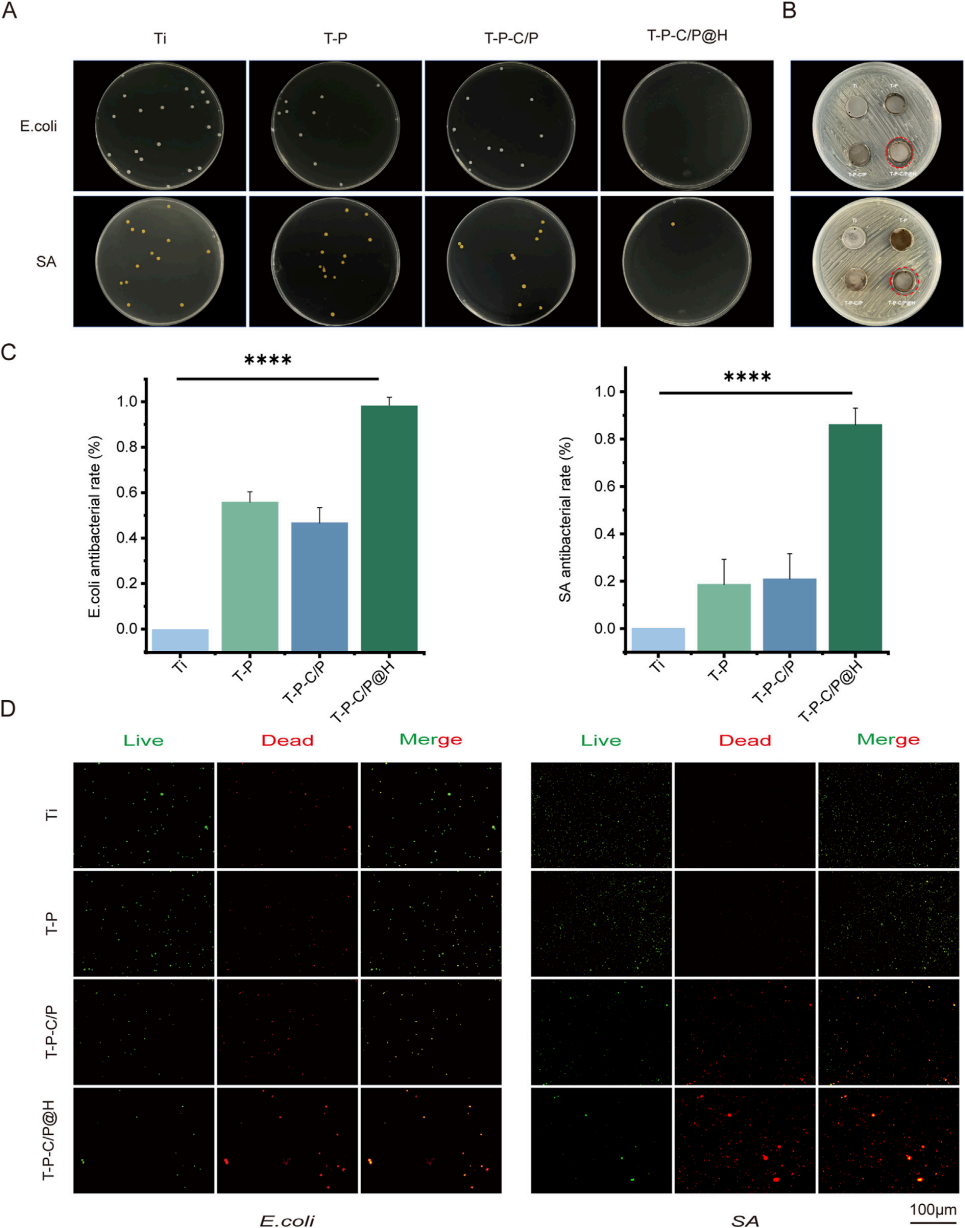
Assessment of the antibacterial ability of T-P-C/P@H *in vitro*. **(A)** The photographs and numbers of bacterial colony formation on agar plates after 24 h of co-culturing titanium materials were treated differently with *E. coli* and SA. **(B)** Photographs of the size of the inhibition zone of *E. coli* and SA on agar plates. **(C)** Contact and release antibacterial rates against *E. coli* and SA of T-P-C/P@H compared with those of the rest 3 groups. **(D)** The live/dead bacterial staining of *E. coli* and SA after 24 h of co-culturing with differently treated titanium materials (green for live bacteria, red for dead bacteria; scale bars = 100 μm). Data are expressed as mean ± SD (n = 3); *p < 0.05, **p < 0.01, ***p < 0.001, ****p < 0.0001, compared with the Ti group.

As shown in Figures 4A, C, the plate count assay results demonstrate that T-P-C/P exhibits moderate antibacterial activity against SA and *E. coli*, primarily attributed to the inherent properties of CS and PDA (Samyn, 2021). However, it fails to completely eliminate the bacteria. In contrast, T-P-C/P@H, through the controlled release of the antimicrobial peptide HHC36, exhibits significant antibacterial effects against both SA and *E. coli*, achieving an inhibition rate exceeding 90%, with statistically significant differences compared to the Ti group. The inhibition zone assay (Figure 4B) further supports this conclusion, indicating that HHC36 may exert stronger antibacterial effects against *E. coli* compared to SA. Additionally, live/dead staining results (Figure 4D) confirm these findings: the Ti group shows intense green fluorescence, indicative of substantial bacterial viability, while T-P-C/P@H exhibits the strongest red fluorescence, signifying bacterial death. T-P-C/P ranks second in antibacterial effectiveness, followed by T-P with minimal red fluorescence. In summary, the release of HHC36 substantially enhances the antibacterial performance of the material. T-P-C/P@H demonstrates excellent antibacterial properties, providing an effective strategy for the surface modification of titanium-based implant materials.

### 3.5 *In vitro* osteogenic ability assessment

#### 3.5.1 ALP

**FIGURE 5:**
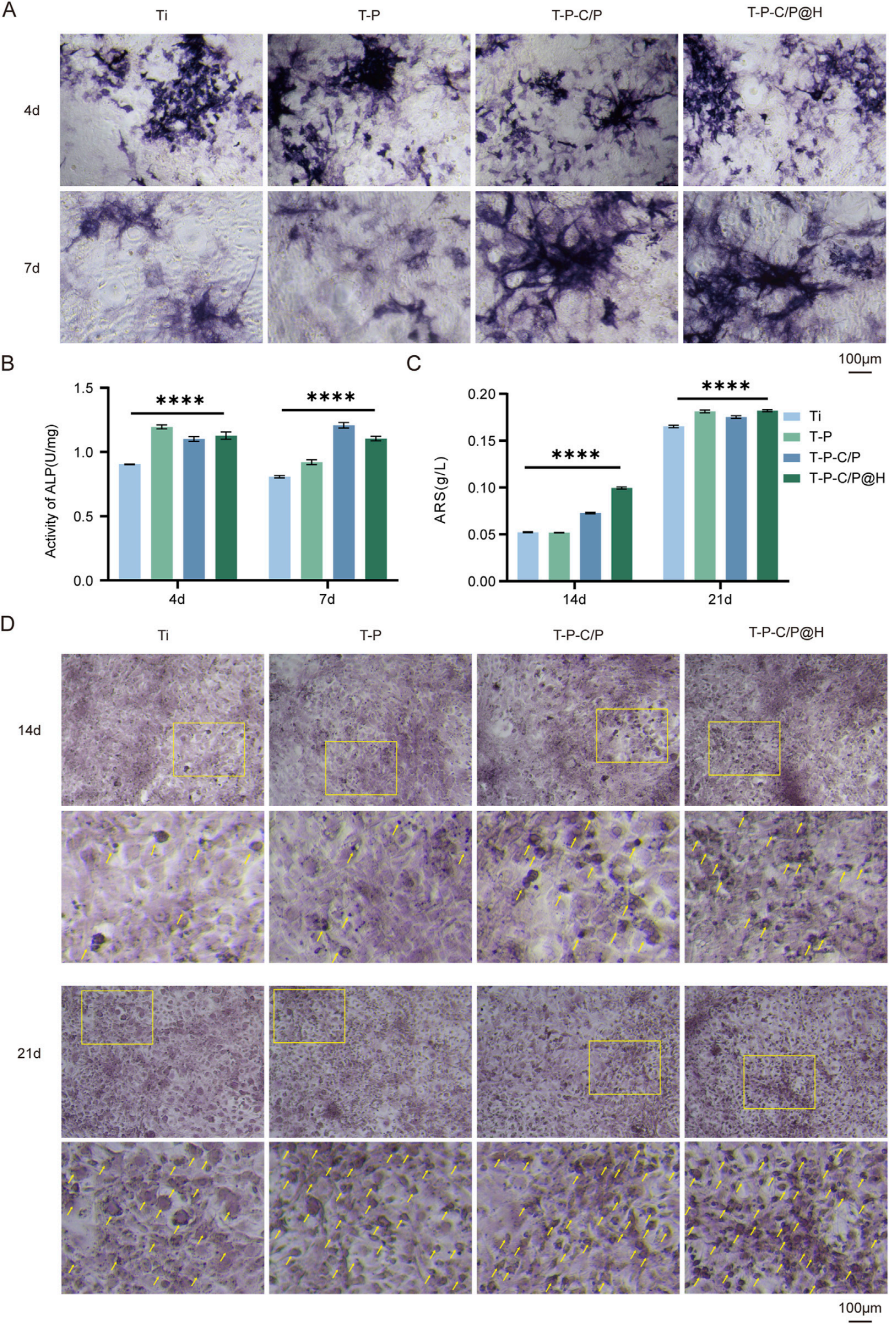
Assessment of the osteogenic ability of T-P-C/P@H *in vitro*. **(A)** Presenting ALP staining after different extract-based treatments following *in vitro* co-culture with MC3T3-E1 for 4 and 7 days (Scale bar = 100 μm). **(B)** Quantitative ALP activity results after different extract-based treatments following *in vitro* co-culture with MC3T3-E1 for 4 and 7 days. **(C)** Quantitative Alizarin Red S staining results after different extract-based treatments following *in vitro* co-culture with MC3T3-E1 for 14 and 21 days. **(D)** Presenting Alizarin Red S staining after different extract-based treatments following *in vitro* co-culture with MC3T3-E1 for 14 and 21 days (Scale bar = 100 μm). Data are expressed as mean ± SD (n = 3); *p < 0.05, **p < 0.01, ***p < 0.001, ****p < 0.0001, compared with the Ti group.

ALP is an early marker of osteoblast differentiation, with its activity significantly increasing during the bone matrix synthesis phase. As shown in Figures 5A, B, on day 4, the ALP activity of T-P, T-P-C/P, and T-P-C/P@H was markedly higher than that of Ti, with T-P exhibiting the highest activity. This is primarily attributed to the higher concentration of PDA in its extract, which promotes osteogenesis (Mahnavi et al., 2022). By day 7, ALP activity in both Ti and T-P showed a slight decrease, while T-P-C/P and T-P-C/P@H maintained elevated levels. This sustained activity may be due to the synergistic effects of PDA, CS, and HHC36. Regardless of whether on day 4 or day 7, ALP activity in Ti remained the lowest, further confirming that all three surface modification methods enhanced osteogenic potential to varying degrees.

#### 3.5.2 Mineralization

As shown in Figures 5C, D, after 14 days of culture, the Alizarin Red staining area increased for the T-P-C/P group and significantly for the T-P-C/P@H group, both markedly higher than that of the Ti. The staining area for the T-P was similar to Ti, indicating that T-P-C/P and T-P-C/P@H effectively enhanced the mineralization and maturation of MC3T3-E1 cells. By day 21, the Alizarin Red staining areas of the T-P, T-P-C/P, and T-P-C/P@H were all higher than that of Ti, with the T-P-C/P@H showing the largest staining area. This suggests that T-P, T-P-C/P, and T-P-C/P@H all have varying degrees of capability to promote the mineralization of MC3T3-E1 cells, with T-P-C/P@H demonstrating the most significant effect, followed by T-P-C/P. At the same time, T-P also shows a relatively good effect.

#### 3.5.3 Detection of osteogenic genes

**FIGURE 6:**
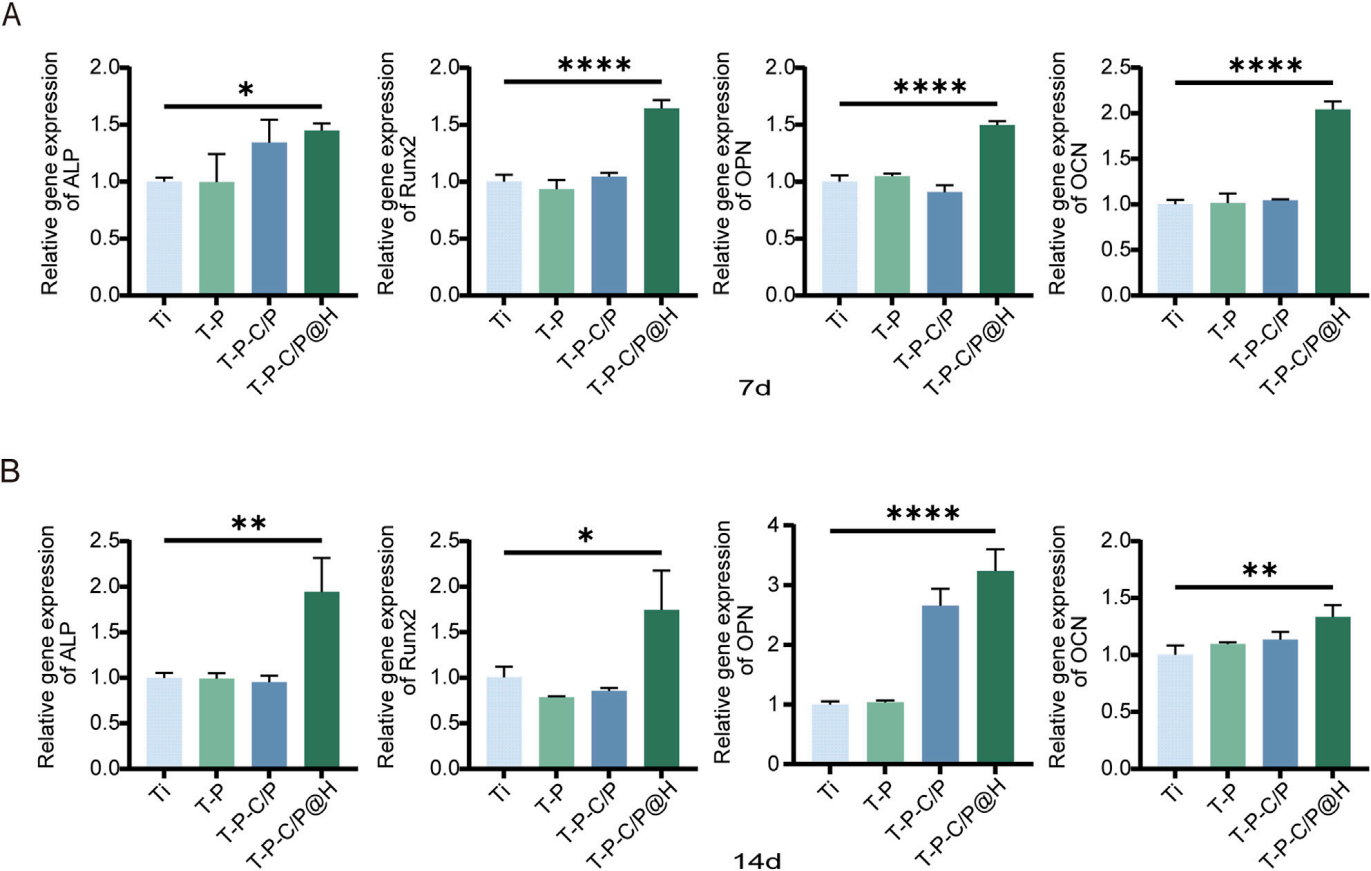
Assessment of the osteogenic ability of T-P-C/P@H *in vitro*. **(A)** Depict the expression levels of osteogenic-related genes (ALP, Runx2, OPN, and OCN) via qPCR analysis after different extract-based treatments following *in vitro* co-culture with MC3T3-E1 for 7 days. **(B)** Depict the expression levels of osteogenic-related genes (ALP, Runx2, OPN, and OCN) via qPCR analysis after different extract-based treatments following *in vitro* co-culture with MC3T3-E1 for 14 days. Data are expressed as mean ± SD (n = 3); *p < 0.05, **p < 0.01, ***p < 0.001, ****p < 0.0001, compared with the Ti group.

This study also assessed the expression levels of several key regulatory factors involved in osteogenic differentiation using qPCR. As shown in Figures 6A, B, the expression levels of all tested genes (ALP, Runx2, OPN, OCN) in T-P-C/P@H increased progressively with longer co-culture times with the material extracts. These levels consistently exceeded those observed in the Ti, T-P, and T-P-C/P groups. Notably, whether at day 7 or day 14, T-P-C/P@H significantly enhanced the expression of osteogenic genes ALP, Runx2, OPN, and OCN, indicating that T-P-C/P@H exhibits relatively stable osteogenic potential. In contrast, the Ti, consisting of pure titanium, served solely to stabilize the implant. T-P and T-P-C/P represent intermediate synthetic stages, where the instability of certain functional groups leads to variability in performance.

## 4 Conclusion

This study successfully developed the T-P-C/P@H composite system by loading CS/PDA@HHC36 hydrogel onto the titanium surface, significantly enhancing the biocompatibility of the titanium substrate and imparting smart pH-responsive antimicrobial properties and osteogenic potential, demonstrating significant clinical application potential. Under normal physiological conditions, the system improves the biocompatibility of titanium-based materials and promotes bone formation, while the hydrophilic barrier formed by the coating helps reduce adverse tissue adhesion. In the presence of infection, the coating can rapidly respond to local pH changes and release HHC36 to combat bacteria. This composite system offers a novel strategy to address both physiological and infection-related challenges, paving the way for the development of efficient implantable medical devices.

## Data Availability

The original contributions presented in the study are included in the article/Supplementary Material, further inquiries can be directed to the corresponding author.
