# Supplementary material for: Construction of pH-responsive hydrogel coatings on titanium surfaces for antibacterial and osteogenic properties
Source: Front Chem. 2025 Feb 19;13:1546637. doi: 10.3389/fchem.2025.1546637 (PMC11883361; doi:10.3389/fchem.2025.1546637)
Supplement: Supplementary file 1 [file DataSheet1.pdf]

# Construction of pH-Responsive Hydrogel Coatings on Titanium Surfaces for Antibacterial and Osteogenic Properties

**Shan Peng[1,2], Yueru Liu[1,2], Wei Zhao[1,2], Xinpeng Liu[1,2], Ronghua Yu[1,2], and Yonglin Yu[1,2*]**

[1]Department of Pathology, Affiliated Hospital of Zunyi Medical University, Zunyi 563003, Guizhou, P. R. China.

[2]Department of Pathology, Zunyi Medical University, Zunyi 563003, Guizhou, P. R. China.

*** Correspondence:**

Corresponding Author: Yonglin Yu

yuyonglin214@163.com

# *Supplementary Material*

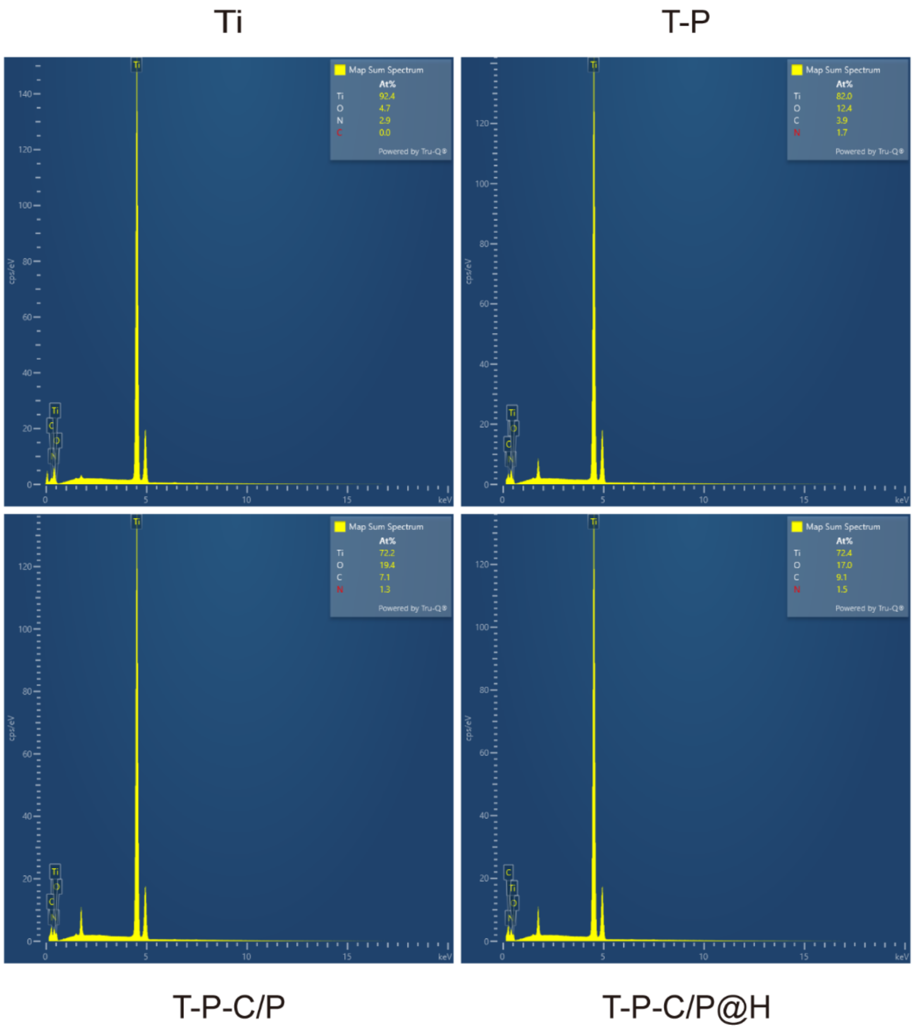


**Figure S1** EDS spectrum for Ti, T-P, T-P-C/P, and T-P-C/P@H.

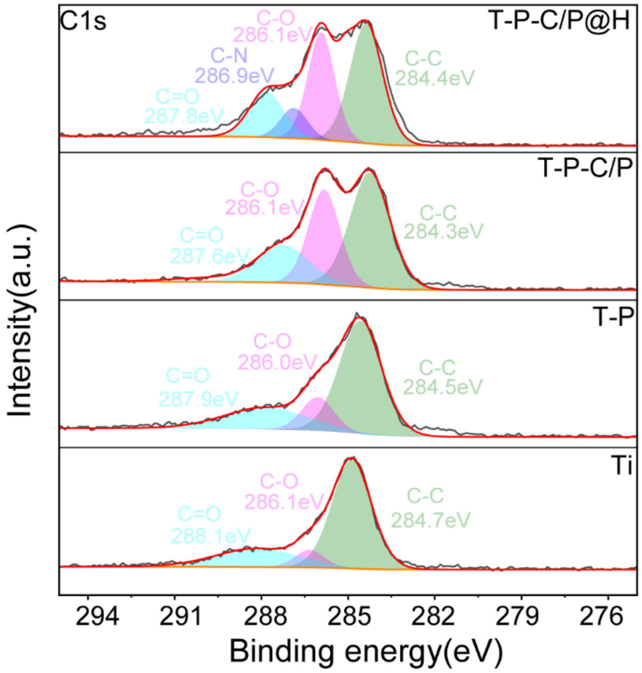


**Figure S2** High-resolution XPS spectra of C1s for Ti, T-P, T-P-C/P, and T-P-C/P@H.

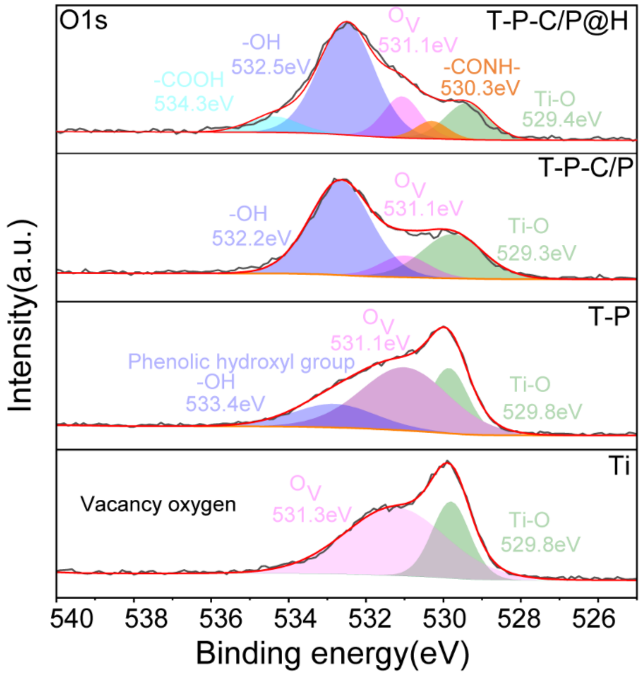


**Figure S3** High-resolution XPS spectra of O1s for Ti, T-P, T-P-C/P, and T-P-C/P@H.

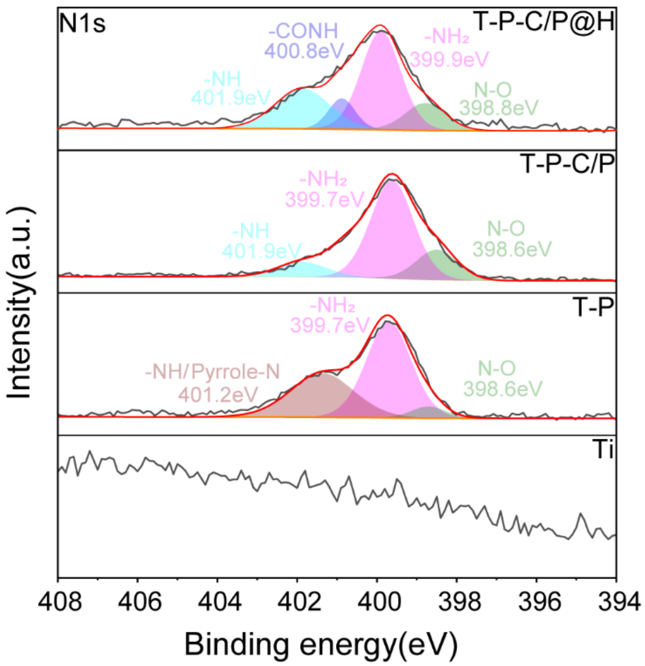


**Figure S4** High-resolution XPS spectra of N1s for Ti, T-P, T-P-C/P, and T-P-C/P@H.
